# High failure rate in meniscal repair when preceding anterior cruciate ligament reconstruction: An analysis of two‐stage surgery for concomitant ACL injury and traumatic meniscus tear

**DOI:** 10.1002/ksa.12593

**Published:** 2025-01-29

**Authors:** Adolfo López Personat, Riccardo Cristiani, Anders Stålman, Johan Wänman, Christoffer Von Essen

**Affiliations:** ^1^ Department of Orthopedics Örebro University Hospital Örebro Sweden; ^2^ Capio Artro Clinic, FIFA Medical Centre of Excellence Sophiahemmet Hospital Stockholm Sweden; ^3^ Department of Molecular Medicine and Surgery, Stockholm Sports Trauma Research Center Karolinska Institutet Stockholm Sweden; ^4^ Department of Surgical and Perioperative Sciences (Orthopedics) Umeå University Umeå Sweden

**Keywords:** ACL reconstruction, knee function, meniscus failure, meniscus repair, staged

## Abstract

**Purpose:**

To investigate the failure rate, predictive factors associated with failure and clinical outcomes after a two‐stage surgery; meniscus repair followed by subsequent anterior cruciate ligament (ACL) reconstruction (ACLR).

**Methods:**

Patients with a concomitant traumatic meniscus tear and ACL injury who underwent a two‐stage surgery between January 2015 and January 2021 were identified. The primary outcome was meniscal repair failure, defined as a reoperation (re‐repair or resection). A Cox‐regression analysis was used in order to analyse factors associated with meniscal repair failure within 3 years after the primary surgery for a meniscal repair. Secondary outcomes were range of motion (ROM), anterior knee laxity and the Knee Injury and Osteoarthritis Outcome Score (KOOS) at 1‐ and 2‐year follow‐up. The thresholds of patient acceptable symptom state (PASS), treatment failure (TF) and minimum important change (MIC) were applied to KOOS4 (mean score of the KOOS Pain, Symptoms, Sports/Rec and QoL subscales).

**Results:**

A total of 150 patients were included. The meniscal repair failure rate after 3 years was 36.7%. Failure of meniscal repair was significantly associated with a time interval >1 year between the meniscal repair to the ACLR (hazard ratio [HR] = 2.5; 95% confidence interval [CI] = 1.2–5.5; *p* < 0.01), medial meniscus repair (HR = 2.3; 95% CI = 1.6–3.4; *P* < 0.01), and female sex (HR = 1.42; 95% CI = 1.0–1.9; *p* = 0.01). The age of the patient was not associated with meniscal repair failure. At the 6‐month follow‐up, most patients (72.5%) showed less than 2 mm of knee laxity; four patients (6.7%) experienced loss of extension and four patients (1.7%) experienced loss of flexion. On the KOOS4, at the 2‐year follow‐up, PASS was achieved in 53.4%, TF occurred in 1.7%, and MIC was reached in 36.4% of patients.

**Conclusion:**

The meniscus repair failure rate after the staged procedure was 36.7% at 3 years. A longer time interval from meniscal repair to ACLR, medial meniscus repair, and female sex were associated with an increased risk of meniscal repair failure. Age was not associated with meniscal repair failure.

**Level of Evidence:**

Level IV, case series retrospective study.

**Abbreviations**
ACL: anterior cruciate ligament
ACLR: anterior cruciate ligament reconstruction
BPTB: bone patellar tendon bone
CI: confidence Interval
HR: hazard ratio
KOOS: Knee injury and Osteoarthritis Outcome Score
LM: lateral meniscus
MIC: minimal important change
MM: medial meniscus
No Reop: no meniscus reoperation
N.S.: not significant
OA: osteoarthritis
PASS: patient acceptable symptom state
Reop: meniscus reoperation
ROM: range of motion
SD: standard deviation
ST: semitendinosus
ST/G: semitendinosus and gracilis
TF: treatment failure

## INTRODUCTION

Adolescents and young adults engaged in pivoting sports have a high incidence of anterior cruciate ligament (ACL) injuries, and approximately one fourth to two thirds suffer from concomitant meniscal injuries [13]. The menisci work as critical knee stabilisers. The medial meniscus (MM) controls anterior tibial translation, whereas the lateral meniscus (LM) restrains rotatory knee laxity [3, 8, 31]. Meniscus repair is associated with more favourable outcomes compared to meniscus resection by restoring the biomechanics in the knee, reducing OA incidence [19, 42], and facilitating return to a high activity level [19]. Despite diligent efforts, the failure rate of meniscal repair within 2–3 years of surgery is not insignificant with a reported incidence between 19% and 36% [32]. Furthermore, in the setting of ACL deficiency, the excessive stress and load caused by abnormal knee laxity limits the meniscus healing and increases the failure rate up to 30%–40% [41]. The context of an ACL‐deficient knee is important as the risk of meniscus repair failure [17], as well as the likelihood of developing cartilage injuries or other new meniscal tears increases for each month postponing ACL reconstruction (ACLR) [1, 13].

The prevailing consensus for the management of meniscus tears in the presence of an ACL‐deficient knee [14, 26] leans towards a single‐stage meniscus repair with concomitant ACLR due to the higher healing rates [25]. Nevertheless, in some cases, surgeons may still opt for a two‐stage surgical approach due to logistical considerations, resource availability, potential concerns about stiffness and loss of range of motion (ROM) or lack of experience in arthroscopic surgery [20, 33].

There is limited data regarding a two‐stage surgical approach for patients with a concomitant meniscus and ACL injury. Majeed et al. [17] reported a higher meniscus repair failure risk when ACLR was performed in a subsequent surgery. On the other hand, Steenbrugge et al. [34] concluded that meniscus repair is not contraindicated in unstable knees. The purpose of this study was to evaluate the meniscal repair failure rate at 3 years from meniscus repair surgery, along with objective (ROM and laxity) and subjective outcomes after two‐stage surgery (meniscal repair followed by subsequent ACLR) at 2 years from ACLR. The hypothesis of this study was that the two‐stage approach would be associated with a high meniscus repair failure rate and might also have a negative impact on objective and subjective outcomes.

## MATERIALS AND METHODS

Ethical approval for this study was obtained from the regional ethics committee (reference no. 2016/1613‐31/32).

### Participants

Patients with a concomitant ACL and a traumatic meniscus tear who underwent a two‐stage surgery (first meniscal repair and subsequently an ACLR) at Capio Artro Clinic, Stockholm, from January 2015 to January 2021 were included in this retrospective cohort study. Patients who previously had an ACLR or meniscus resection or repair in the same knee were excluded. There were no bi‐meniscal lesions in the included patients. All the meniscus injuries had a traumatic etiology [14], characterised by sudden onset of symptoms resulting from significant trauma.

### Outcome variables

Data on sex, age at the time of the primary surgery, associated injuries, Tegner activity level [36] and occurrence of meniscus failure defined as meniscus reoperation, either a resection or a new repair, and clinical outcomes were collected from a local database (Table 1).

**Table 1:** Patient characteristics and surgical data (*n* = 150 patients).

Age at primary surgery (years)	Mean ± SD	28.0 ± 10.5
Gender (female)	*n* (%)	79 (52.7)
Tegner activity level pre‐injury	Median (range)	7 (3–10)
Time injury‐surgery (days)	Median (range)	26 (1–963)
Time meniscus repair‐ACLR (days)	Median (range)	106 (31–1074)
ST/STG	*n* (%)	112 (74.4)
BPTB	*n* (%)	8 (5.3)
Quad‐tendon	*n* (%)	30 (20.0)
Medial meniscus repair at primary surgery	*n* (%)	123 (82.0)
Medial Meniscus repair at ACLR	*n* (%)	33 (21.9)
Medial Meniscus resection at ACLR	*n* (%)	23 (15.3)
Medial meniscus re‐repair	*n*	24
Lateral meniscus repair at primary surgery	*n* (%)	27 (18.0)
Lateral Meniscus repair at ACLR	*n* (%)	19 (12.6)
Lateral Meniscus resection at ACLR	*n* (%)	8 (5.3)
Lateral meniscus re‐repair	*n*	6
Cartilage injury at ACLR	*n* (%)	20 (13.3)

Abbreviations: ACLR, anterior cruciate ligament reconstruction; BPTB, bone patellar tendon bone; Quad, quadriceps; SD, standard deviation; ST, semitendinosus; STG, semitendinosus and gracilis.

### Surgical technique and post‐operative rehabilitation

Surgical technique was based on the surgeon's preference. Standard arthroscopic procedures with anteromedial and anterolateral portals were used. Surgeries were performed by a total of 22 surgeons, all of them classified as high‐volume [24], with ≥50 procedures annually, including at least 29 ACLRs or revisions per year. Meniscal repair was performed with an all‐inside technique using either FastFix (Smith & Nephew, Andover) or Fiberstitch suture anchor devices (Arthrex) for tears located in the posterior horn or corpus of the menisci. If the tear required an outside‐in or inside‐out repair technique, a standard medial or lateral incision was used, and the tear was repaired with a PDS 0 (Ethicon). The number of sutures used was decided by the surgeon, with the tear repaired until stability was confirmed through probe testing. ACLRs were performed using a single bundle technique. Hamstring, bone‐patellar tendon‐bone, or quadriceps tendon autografts were used. Hamstring autografts (quadrupled semitendinosus tendon [ST] or ST and gracilis tendon [ST/G]) were the most frequently used grafts, accounting for 74.7% of the cases. After surgery, weight‐bearing was encouraged as tolerated and patients wore a hinged knee brace for a total of 6 weeks, gaining 30° of flexion every 2 weeks (0–30° first and second, 0–60° third and fourth and 0–90° fifth and sixth weeks). All patients adhered to a standardised post‐operative rehabilitation protocol and were instructed to avoid deep squatting for 4 months after meniscus repair.

### Patient evaluation

ROM and knee laxity were measured preoperatively and at 6 months after the ACLR. Loss of extension or flexion was defined as a difference in extension or flexion, respectively, >5° compared to the contralateral healthy knee. ROM was measured with a goniometer. The KT‐1000 arthrometer (MEDmetric) with a standard anterior tibial load of 134 N at 20° of knee flexion was used to measure anterior tibial translation. The side‐to‐side difference was registered. All measurements were performed by experienced physical therapists. The Knee injury and Osteoarthritis Outcome Score (KOOS) was collected preoperatively and at 1‐ and 2‐year follow‐up. The KOOS evaluates patients' perception of knee function on five subscales (*Pain*, *Symptoms*, *Activities of Daily Living*, *Sports* and Recreation and *Quality of Life*), and scores range from 0 (*worst*) to 100 (*best*) [28]. Analyses were made using KOOS4 (mean score of KOOS Pain, Symptom, Sports and Recreation, and Quality of Life subscales) [7]. To study the clinical relevance of patients' experiences of improvement after ACLR and evaluate whether acceptable outcomes have been achieved, three key thresholds are often applied: Minimal Important Change (MIC), patient acceptable symptom state (PASS) and treatment failure (TF). MIC threshold is used to identify the minimal change that patients would consider important to reach after the intervention; [5] PASS indicates patients who consider themselves well [37], and TF is used to mark participants who felt their treatment has failed [10]. The threshold [4] values for MIC, PASS and TF from the Roos et al. study [27] were applied to the 1‐ and 2‐year KOOS4 follow‐up assessments [22].

### Statistical analysis

Statistical analysis was performed with SPSS (version 25.0) software package. Continuous variables were reported as mean with standard deviation (SD) if they were normally distributed or otherwise as medians with range. Categorical variables were reported as numbers (*n*) and proportions (*%*). Factors associated with meniscal repair failure within 3 years of primary surgery (meniscal repair) were analysed with a Cox regression model. Age at first surgery, gender of the patients, laterality of the meniscus, and time interval from meniscus repair to ACLR surgery were used as variables. The results were reported as hazard ratios (HRs) with corresponding 95% confidence intervals (CIs). A *p* value < 0.05 was considered statistically significant.

## RESULTS

A total of 150 participants were included in the study, representing a mixed population with diverse activity levels. The median pre‐injury Tegner activity level was 7 (range 3–10). The median time from injury to meniscus repair was 26 days (range 1–963) and the median time from meniscal repair to ACLR was 106 days (range 31–1074). None of the patients underwent concomitant medial and lateral meniscal repair during the first procedure, as there were no bi‐meniscal lesions. During the first procedure, MM repair was performed in 82% of cases, whereas LM repair was performed in 18% of cases. The study population and surgical data of the patients included are presented in Table 1.

### Meniscal repair failure

**Figure 1:**
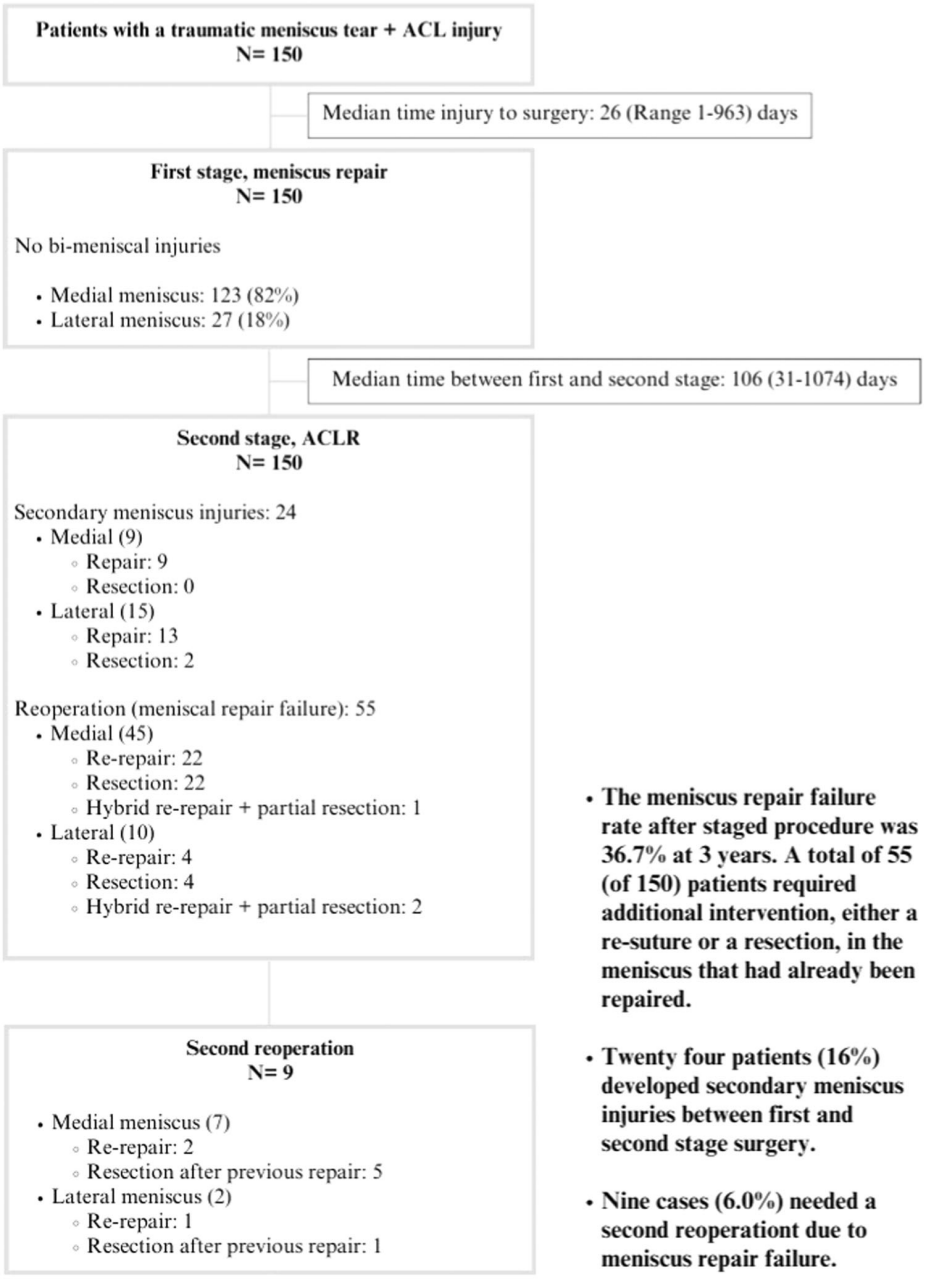
Flow chart: meniscus repair failure. The meniscus procedures performed at the primary and secondary stages, as well as during the second reoperation are presented. The time between injury and the procedures is reported as the mean number of days and range. ACL, anterior cruciate ligament; ACLR, anterior cruciate ligament reconstruction.

During the second stage of surgery (ACLR), 55 patients required additional intervention due to meniscus repair failure. Among these cases, 45 involved the MM, while 10 involved the LM. These interventions included re‐repair, meniscectomy or hybrid repair (combined repair with partial resection). In addition, 24 patients (16%) developed new, secondary meniscus injuries during the interval between surgeries, which were not present during the first stage of surgery. These secondary meniscus injuries were treated during the ACLR procedure. Figure 1 summarises the meniscus procedures, their laterality and the reoperations performed.

At the 3‐year follow‐up, 55 out of 150 cases (36.7%) were reported as meniscus repair failures. Furthermore, nine patients (6.0%) required a second reoperation on a previously treated meniscus in a third surgery.

### Factors associated with meniscal repair failure

Female gender (hazard ratio [HR] = 1.42; 95% confidence interval [CI] = 1.1–1.9; *p* = 0.01), MM repair (HR = 2.3; 95% CI = 1.6–3.4; *p* < 0.01), and time interval longer than 1 year between meniscal repair and ACLR (H = 2.5; 95% CI= 1.2–5.5; *p* < 0.01) were associated with a significantly increased hazard of meniscal repair failure at the 3‐year follow‐up in the Cox regression analysis. Age was not associated with meniscal repair failure, Table 2.

**Table 2:** Factors associated with meniscus repair failure in the multivariate Cox regression.

	Multivariate Cox regression		
Variable	HR	95% CI	*p*
Gender			
Male (ref)		1	
Female	1.42	1.1–1.9	0.013
Age, years			0.7
<20 (ref)		1	
21–30	1.1	0.8–1.5	0.2
31–40	1.0	0.6–1.5	0.4
>40	1.0	0.7–1.6	0.3
Meniscus			
Lateral (ref)		1	
Medial	2.3	1.6–3.4	<0.001
Days meniscus‐repair to ACLR			0.02
0–90 (ref)		1	
91–180	0.7	0.3–1.5	0.4
181–365	1.1	0.5–2.5	0.8
>365	2.5	1.2–5.5	0.01

Abbreviations: ACLR, anterior cruciate ligament reconstruction; CI, confidence interval; HR, hazard ratio; ref, reference level.

### Patient‐reported outcomes

**Figure 2:**
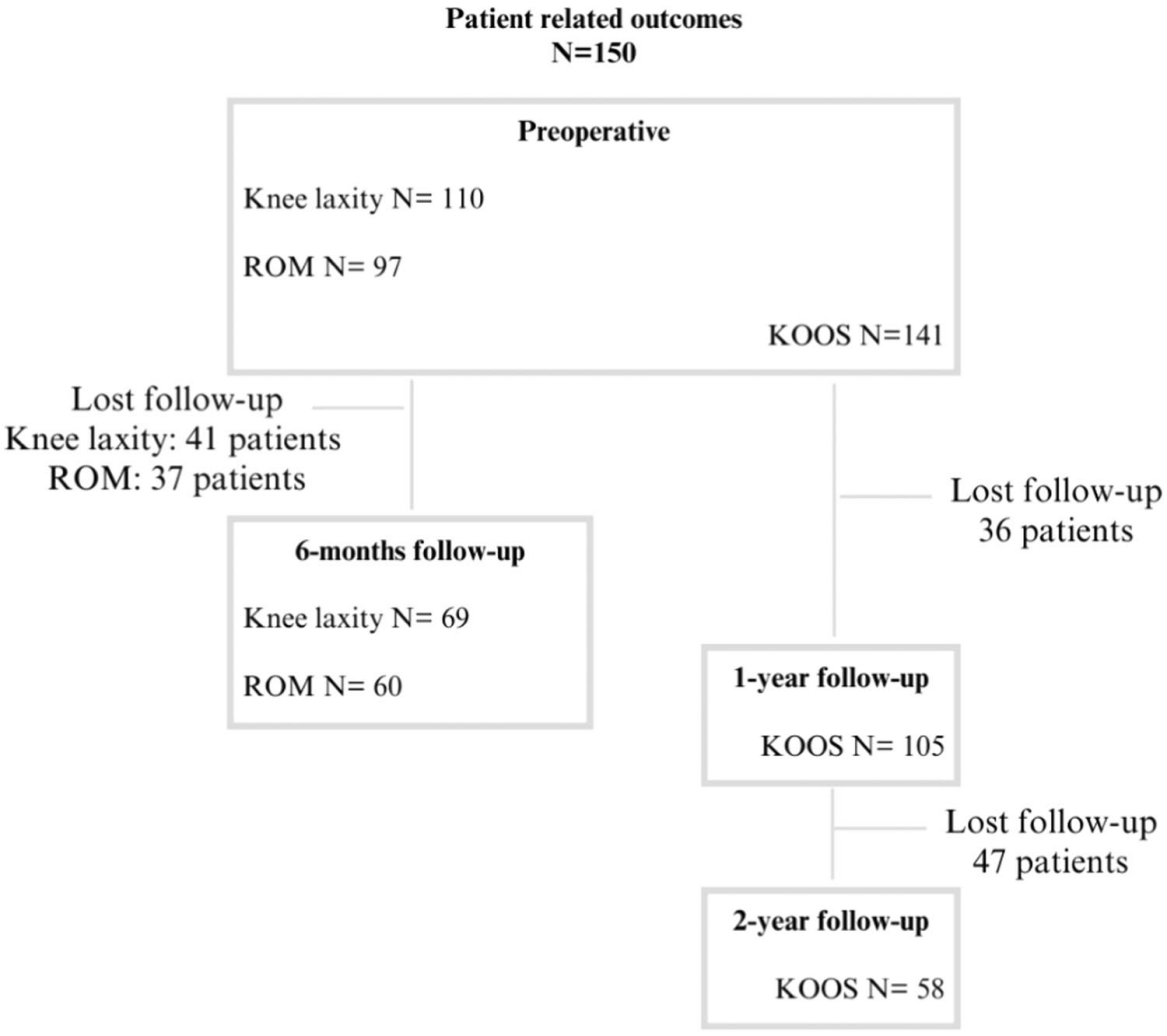
Flow chart: patient‐related outcomes. The available data on knee laxity, range of motion (ROM) and Knee Injury and Osteoarthritis Outcome Score (KOOS) at different follow‐up points, as well as the corresponding lost‐to‐follow‐up, are represented.

**Figure 3:**
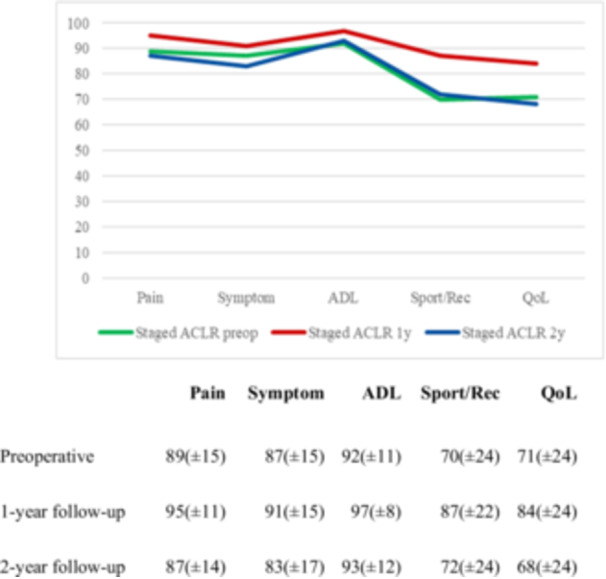
Overall KOOS preoperative, 1‐year and 2‐year follow‐up. ADL, activities of daily living subscale; KOOS, Knee Injury and Osteoarthritis Outcome Score; Sport/rec, sport‐recreation.

Data collection flow chart for knee laxity, ROM and KOOS at the different follow‐up intervals, with the corresponding number of patients lost to follow‐up for each outcome, is presented in Figure 2. Two‐year follow‐up for the KOOS was obtained for 58 patients (38.7%). There were no significant differences in the patient‐reported outcomes between patients with or without a meniscal repair failure. The overall KOOS is reported in Figure 3, while the number of patients that reached the PASS, MIC, and TF threshold at 1‐ and 2‐year follow‐ups are presented in Table 3. The KOOS for patients who underwent reoperation on the meniscus and those who did not is presented in Table 4. Six months of ROM follow‐up was performed in 60 patients (40%). Before surgery, five patients (5.2%) had a loss of extension, compared to four patients (6.7%) at the 6‐month follow‐up. In terms of flexion, four patients (6.7%) experienced a loss preoperatively, while one patient (1.7%) had a loss at the 6‐month follow‐up. For knee laxity, 6‐month follow‐up was performed in 69 patients (46%); 61.8% of cases had a side‐to‐side difference of 2 mm or less preoperative, compared to 72% at the 6‐month follow‐up. There were no significant differences in the ROM and knee laxity between patients with or without a meniscal repair failure (Table 5).

**Table 3:** Proportion of patients achieving PASS, TF and MIC at 1‐year and 2‐year follow‐up.

1‐year follow‐up	*n* (%)	PASS (KOOS4)		TF (KOOS4)		MIC (KOOS4)	
	*n* = 105 (70.0)	79 (75.2)		4 (3.8)		33 (32.7)	
Reop	36 (34.3)	26 (72.2)	n.s.	2 (5.6)	n.s.	11 (30.6)	n.s.
No reop	69 (65.7)	53 (76.8)		2 (2.9)		22 (31.9)	

2‐year follow‐up	*n* (%)	PASS (KOOS4)		TF (KOOS4)		MIC (KOOS4)	
	*n* = 58 (38.7)	31 (53.4)		1 (1.7)		20 (36.4)	
Reop	23	13 (56.5)	n.s.	1 (4.3)	n.s.	7 (29.2)	n.s.
No reop	35	18 (51.4)		0		13 (37.1)	

Abbreviations: KOOS4, mean score of the Knee injury and Osteoarthritis Outcome Score Pain, Symptoms, Sports/Recreation, and Quality of Life subscales; MIC, minimal important change; n.s., not significant; No Reop, no meniscus reoperation; PASS, patient‐acceptable symptom state; Reop, meniscus reoperation; TF, treatment failure.

**Table 4:** KOOS preoperative, 1‐year and 2‐year follow‐up for patients who underwent meniscus reoperation compared to those who did not.

	Pain	Symptom	ADL	Sport/Rec	QoL
No reop					
Preoperative	92 ± 13	91 ± 16	97 ± 7	75 ± 24	76 ± 24
1‐year follow‐up	95 ± 9	92 ± 15	94 ± 10	88 ± 22	85 ± 24
2‐year follow‐up	87 ± 13	82 ± 16	93 ± 12	72 ± 24	67 ± 23
Reop					
Preoperative	88 ± 17	87 ± 17	92 ± 11	67 ± 24	71 ± 24
1‐year follow‐up	93 ± 13	90 ± 15	96 ± 10	80 ± 22	83 ± 24
2‐year follow‐up	87 ± 16	83 ± 17	92 ± 15	70 ± 24	68 ± 24

*Note*: Data for the KOOS subcategories are presented as mean ± standard deviation.

Abbreviations: ADLs, activities of daily living subscale; KOOS, Knee injury and Osteoarthritis Outcome Score; QoL, quality of life; Sport/rec, sport‐recreation; no reop, no meniscus reoperation; Reop, meniscus reoperation.

**Table 5:** Knee laxity (KT‐1000 arthrometer) and range of motion (ROM) measurements at 6‐month follow‐up.

Knee laxity		Reop	No Reop		ROM		Reop	No reop	
Preop	*n* = *110*	*40*	*70*		Preop	*n* = *97*	39	58	
<2 mm	68 (61.8)	26 (65.0)	42 (60.0)		Loss of extension	5 (5.2)	1 (2.6)	4 (6.9)	n.s.
2–5 mm	28 (25.5)	10 (25.0)	18 (25.7)		Loss of flexion	4 (4.1)	1 (2.6)	3 (5.2)	n.s.
>5 mm	14 (12.7)	4 (10.0)	*10* (*14.3)*		6m	*N* = *60*	25	35	
6m	*n* = *69*	*26*	43	n.s.	Loss of extension	4 (6.7)	1 (4)	3 (8.6)	n.s.
<2 mm	50 (72.5)	18 (69.2)	33 (76.7)	n.s.	Loss of flexion	1 (1.7)	0	1 (2.9)	n.s.
2–5 mm	18 (26.1)	8 (30.8)	9 (20.9)						
>5 mm	1 (1.4)		1 (2.3)						

*Note*: Loss of extension or flexion was defined as a difference (in extension or flexion, respectively) >5° compared with the contralateral healthy knee

Abbreviations: no reop, no meniscus reoperation; Preop, preoperative; Reop, meniscus reoperation.

## DISCUSSION

The most important finding was that patients who underwent two‐stage surgery had a high meniscal repair failure rate of 36.7% at 3 years. The factors associated with meniscal repair failure were female sex, MM repair, and a longer time interval between meniscal repair and ACLR. Meniscal repair failure did not adversely affect knee laxity, range of motion, or patient‐reported outcomes at 2‐year follow‐up.

The basis for a successful meniscal repair is solid mechanical fixation, adequate stability and an effective biological healing process. A recent meta‐analysis [32] reported an overall meniscus repair failure rate of 14.8%. Interestingly, subgroup analysis revealed a significantly lower failure rate for meniscal repair performed concurrently with ACLR compared to isolated meniscus repair in knees without reported ACL injury (8.5% vs. 14%, *p* = 0.043). In contrast, previous studies analysing meniscus repair failure in ACL‐deficient knees have reported failure rates between 22% and 45% [17, 18, 20, 41]. Warren et al. [41] reported a 30%–40% meniscus repair failure rate in knees that did not undergo ACLR. O'Shea [20] et al. reported that at the time of the ACLR (in the mean 77 days after meniscus repair of bucket handle meniscus tear), 55% of menisci appeared healed, while 45% required additional procedures. These findings are similar to the failure rate in the present study, suggesting that the instability of the ACL‐deficient knee may adversely affect meniscus healing. The ACLR environment may promote potential biological benefits; studies have shown that the release of growth factors during the drilling of the femoral and tibial tunnel can significantly enhance meniscal healing rates [16, 23]. In contrast, a recent meta‐analysis by Nepple et al. [19] found no difference in meniscus repair failure between ACL‐intact and ACL‐deficient knees, however, only three studies (with a small study population) allowed for a direct comparison. Malinowski et al. [18], reported a 75.6% complete healing rate in meniscus repairs assessed during ACLR that was performed 6–8 weeks after meniscus repair. Majeed et al. [17] observed a meniscus repair failure rate of 27% in the subgroup that underwent ACLR after an initial meniscal repair and after 6 weeks of injury. These findings suggest that the time period between meniscus repair and ACLR, which was longer in the present study (median: 106 days) may be a crucial factor. An increased risk of meniscus repair failure was noted when ACLR was delayed for more than one year from the meniscus repair. This risk may stem from the same mechanisms that cause new meniscal and chondral injuries in ACL‐deficient knees over time [1, 14, 23]. In the present study, 29% (44 out of 150) of patients developed new meniscal injuries during ACLR that were not present at the initial meniscus repair. This highlights the importance of timely ACLR, to reduce the risk of further injuries and to promote effective meniscus healing. It is possible that the decision to opt for a staged procedure was motivated by the presence of more extensive meniscal tears which required prompt repair. If so, the likelihood of successful meniscal healing could have been affected by the injuries' initial severity.

In this study, meniscal laterality was a significant factor for reduced meniscus repair failure rate. This is consistent with previous reports in the literature. Schweizer et al. [32] concluded in their meta‐analysis that the failure rate for lateral meniscal repairs was significantly lower than that for medial meniscal repairs (6.1% vs. 10.8%, *p* = 0.031). Similarly, Rönnblad et al. [29] reported a fourfold lower risk of reoperation after LM repair compared to MM repair. The higher failure rates for MM repairs may be attributed to the increased mechanical stress on this structure in the ACL‐deficient knee, where it becomes the primary restraint of anterior laxity [31]. Moreover, the LM has increased mobility during knee motion [8], this may help reduce stresses on the repair. Furthermore, differences in vascularisation between the menisci also affect healing rates [6, 21]. The MM repairs in this research showed more than two times HR for failure compared to LM. Given that 82% of repairs in the present study were performed on the MM, this distribution may have influenced the overall meniscal repair failure rate.

The general consensus in the literature suggests that patient sex does not significantly influence the risk of meniscus repair failure. A recent systematic review by Hamilton et al. [9] found no significant gender differences in meniscus repair outcomes, with only 1 of the 11 included studies reporting a difference in failure risk based on sex. That study, conducted by Zimmerer et al. [43], showed a higher risk of meniscal repair failure in females. Its findings, however, were limited by a small sample size and a low follow‐up rate of only 19.4%. In the present study, Cox regression analysis revealed that female patients had an HR of 1.42 (95% CI = 1.1–1.9; *p* = 0.01) for meniscus repair failure compared to male patients. The observed differences cannot be explained. Further research would be necessary to better understand the potential gender‐related variations in meniscus repair outcomes.

Age has traditionally been seen as a negative factor for meniscal repair due to concerns about chronic, degenerative tears with low healing potential. A recent systematic review has found no increased risk of reoperation with age [12]. Similarly, no association between age and increased meniscal repair failure rates was found in this study. This may be explained by the patients' lower physical activity levels or the surgeon's more cautious selection of cases for both the initial repair and any subsequent reoperation.

Meniscal repair is paramount to reducing the risk of OA development, improving long‐term patient outcomes and restoring knee kinematics [42]. A failed meniscal repair not only increases the risk for OA but is also associated with an inferior subjective knee function in the long term [30]. Medial meniscectomy is a factor associated with an increased risk of abnormal anterior knee laxity after primary ACLR [4]. MM resection was performed in 28 patients (18.7%) in the current research. This could affect overall knee laxity measurements due to the MM's role in restraining anterior tibial translation [4]; however, the extent of the meniscectomy can vary significantly, from minor trimming to total meniscectomy. Knee laxity measurements at 6‐month follow‐up were performed in 69 patients (46%); 50 of them (72.5%) had a normal (difference side to side of less than 2 mm), and only 1 patient (1.4%) had a difference higher than 5mm.

Some authors who have advocated for a staged procedure highlight improved ROM recovery as the main advantage [31, 33]. Two recent studies on two‐stage surgery outcomes [17, 18] did not specifically assess ROM. Furthermore, recent research indicates no significant ROM issues with concomitant ACLR and meniscus surgery, even in acute ACLR cases [40]. A recent study has shown promising meniscus survivorship and satisfactory patient‐reported outcomes following single‐stage meniscus bucket‐handle repair combined with ACLR, even in cases where the meniscus had been displaced for an extended period [25]. In the present study, only five patients experienced ROM loss at the 6‐month follow‐up: four patients (6.7%) had a loss of extension, and one patient (1.7%) had a loss of flexion.

There is on‐going debate regarding how meniscal repair or resection impacts short‐term KOOS [2, 35]. A study analysing a two‐stage procedure with 6–8 weeks between meniscus repair and ACLR found that even with incomplete signs of meniscal healing, 66.7% demonstrated favourable clinical self‐reported outcomes at the 24‐month follow‐up [18]. In the present study, 2‐year follow‐up KOOS data was obtained for 58 patients (38.7%). Slightly inferior outcomes were reported in three categories compared to preoperative scores (Pain: 87 vs. 89, Symptom: 83 vs. 87, Qol: 68 vs. 71), with minimal improvement in Sport/Recreation (72 vs. 70) and ADL (93 vs. 92). Those results align with previous research that did not find worse patient‐related outcomes after meniscus revision [15]. When interpreting the results, 53.4% of patients felt good (PASS), 36.4% felt better (MIC) and only 1.7% considered themselves treatment failures. No differences in KOOS were shown in patients who underwent meniscus reoperation. von Essen et al. [39] found that patients who had additional surgeries within 2 years of their primary ACLR reported worse outcomes. In the current study, the fact that meniscus reoperation was performed at the time of ACLR (and not as an additional intervention) may have influenced patients' subjective perceptions. Jacksson et al. [13], reported in their systematic review that revision meniscus repair failure in patients with re‐tears or non‐healed meniscus results in clinical outcomes similar to primary repairs.

The findings of this study are relevant for clinical practice, providing insights for decision‐making regarding meniscus preservation. There is a knowledge gap in the literature about outcomes after two‐staged meniscus surgery, moreover, patients in those studies follow different rehabilitation protocols and restrictions, and there is a high variability in the meniscus tears analysed, complicating comparisons between the studies [17, 18, 20, 33]. Definitions of meniscus failure also vary [9, 11, 12, 13, 14, 15, 17, 19, 25, 29, 30, 32, 43]. Although some authors [18, 20, 33] supported the two‐stage approach, surgeons should take into consideration the high risk of meniscal repair failure in ACL‐deficient knees. Meniscus repair with concomitant ACLR should be the first option [14]; if other logistical factors come into play and the two‐stage procedure is the only viable option, it is recommended to perform the subsequent ACLR as soon as feasible and in any case with a longer delay than 1 year.

We did not find support for the hypothesis that secondary outcomes would worsen due to the staged approach and the increased meniscus repair failure rate. However, those results should be analysed with caution due to significant follow‐up loss. Addressing long‐term outcomes could be interesting in future research.

Strengths of this study include a large sample size and the involvement of different high‐volume surgeons, enhancing generalisability. Another strength was the standardised rehabilitation protocol. This study has several limitations. The main limitation is the retrospective design. A further limitation is the fact that injuries to the meniscus and ACL may have varied in terms of chronicity and the lack of this data could potentially affect both meniscal repair survival and clinical outcomes. Similarly, specific data regarding the characteristics of the meniscal tears was not obtained, with no available information regarding the type or extension of the tear or the specific location of the injury in the Cooper zones, which may influence failure rates [21, 38]. Another limitation was the absence of a control group. This study had missing follow‐up affecting secondary outcomes, furthermore, only short‐term patient‐reported outcomes are analysed. Reasons that prompted surgeons to opt for a staged procedure are not included in the study; theoretically, these patients may have had more severe, urgent or complex injuries, introducing a selection bias.

## CONCLUSION

The meniscus repair failure rate after the staged procedure was 36.7% at 3 years. A longer time interval from meniscal repair to ACLR, MM repair and female sex were associated with an increased risk of meniscal repair failure. Age was not associated with meniscal repair failure.

## AUTHOR CONTRIBUTIONS

Adolfo López Personat: Drafting of the manuscript, participation in the study design, data interpretation and design of tables and figures. Author of the final version. Riccardo Cristiani: collection of patient data, interpretation and database management. Final approval. Anders Stålman: review of the manuscript, data interpretation and support in study design. Final approval. Johan Wänman: review of the manuscript, data interpretation participation in study design. Final approval. Christoffer Von Essen: study design, data collection, statistical analysis, data interpretation, database management, tables and figures design and manuscript review. Final approval.

## ETHICS STATEMENT

Ethical approval for this study was obtained from the regional ethics committee at Karolinska Institute (reference no. 2016/1613‐31/32).

## CONSENT

All patients provided informed consent for participation in the study and for the use of their clinical data for research purposes. The consent process adhered to the ethical guidelines established by the approving ethics committee.

## CONFLICTS OF INTEREST STATEMENT

The authors declare no conflicts of interest.

## Data Availability

Study data were extracted from the local database at Capio Artro Clinic, Stockholm.
